# P-316. Multi-Drug Resistant Organism Colonization is Dynamic and Associated with Adverse Outcomes in Critically Ill Patients

**DOI:** 10.1093/ofid/ofae631.519

**Published:** 2025-01-29

**Authors:** Max W Adelman, Andrea M Detranaltes, Jiaqiong Xu, Giselle Ortiz, Marissa G Schettino, Abigail A Amaya, Husna Malikzad, Muhammad H Virk, Asmita Ghosh, Roberta Higgins, Shubhra Singh, Kirsten Rydell, Mary N Jones, Rachel Atterstrom, Blake M Hanson, Rodrigo de Paula Baptista, Samuel A Shelburne, Tor Savidge, Cesar A Arias

**Affiliations:** Houston Methodist Hospital, Houston, Texas; Houston Methodist Hospital, Houston, Texas; Houston Methodist Research Institute, Houston, Texas; Houston Methodist Hospital, Houston, Texas; Houston Methodist Hospital, Houston, Texas; Houston Methodist Hospital, Houston, Texas; Houston Methodist Hospital, Houston, Texas; Houston Methodist Hospital, Houston, Texas; Houston Methodist Hospital, Houston, Texas; Houston Methodist Hospital, Houston, Texas; Houston Methodist Hospital, Houston, Texas; Houston Methodist Hospital, Houston, Texas; Houston Methodist Hospital, Houston, Texas; Houston Methodist Hospital, Houston, Texas; The University of Texas Health Science Center, Houston, Texas; Houston Methodist Hospital, Houston, Texas; MD Anderson-University of Texas, Houston,, Texas; Baylor College of Medicine, Houston, Texas; Houston Methodist and Weill Cornell Medical College, Houston, TX

## Abstract

**Background:**

Critically ill patients have frequent colonization with multi-drug resistant organisms (MDRO). Colonization is assumed to be a static state, but intensive care unit (ICU) patients may be exposed to various interventions that affect their colonization status; this has not been rigorously examined.

**Table 1.**
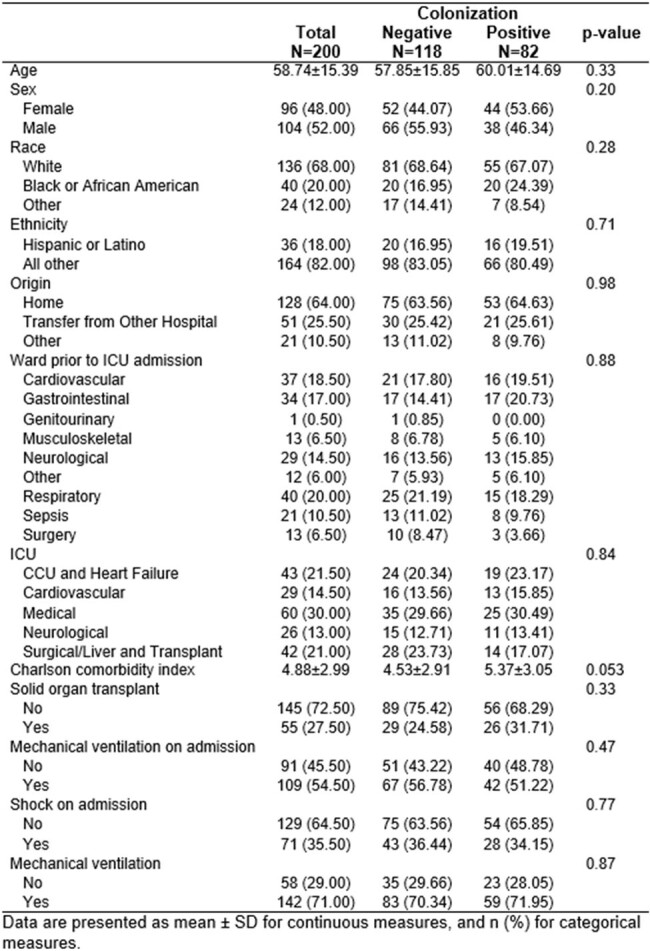
Baseline characteristics of intensive care unit patients by multidrug-resistant stool colonization status.

**Methods:**

We performed a prospective cohort study of adult ICU patients. Patients had stool samples collected twice weekly for ≤ 4 weeks or until ICU discharge; samples were plated on selective media for the MDROs vancomycin-resistant enterococci (VRE), extended-spectrum β-lactamase-producing Enterobacterales (ESBL-E), and carbapenem-resistant Enterobacterales (CRE). We characterized patient demographics and changes in colonization status and compared outcomes between patients with and without MDRO colonization using a desirability of outcomes ranking (DOOR) analysis, which considered three outcome levels (alive, alive with infection, or dead).

**Figure 1.**
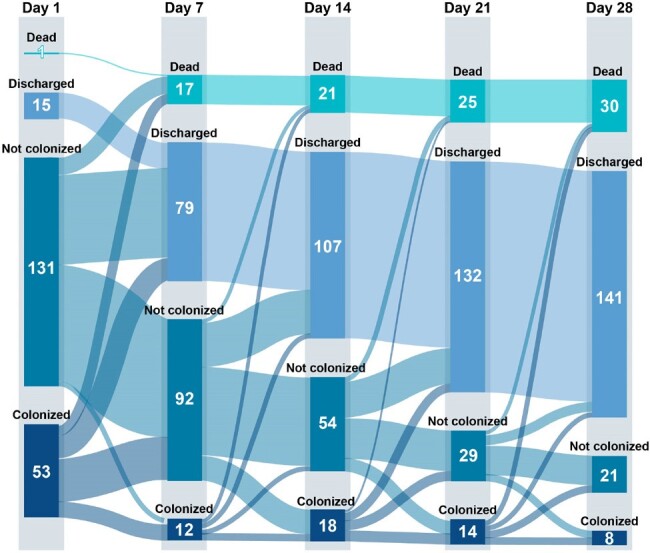
Changes in multi-drug resistant stool colonization status over time for 200 patients admitted to an intensive care unit.

**Results:**

We included 200 patients; 82 (41%) were colonized at ≥1 time point; 50 (25%) had ≥ 50% of their samples colonized; 32 (16%) had ≥ 2 consecutive samples with colonization (“persistent”). There were no differences in baseline characteristics between patients with and without any colonization (**Table 1**), or between patients with or without ≥ 50% or persistent colonization. We observed flux in colonization status (**Figure 1**); for example, of 53 patients colonized on day 1, 25 (47%) were not colonized on day 7. Of 119 patients with multiple samples, 38 (32%) had changes in their colonization status from first to last sample (**Figure 2**). Any colonization or ESBL/CRE colonization was not associated with adverse outcomes. However, patients with VRE colonization had worse DOOR outcomes than patients without (58.4% probability of a worse DOOR outcome, 95% CI 50.3%-66.2%) (**Figure 3**).

**Figure 2.**
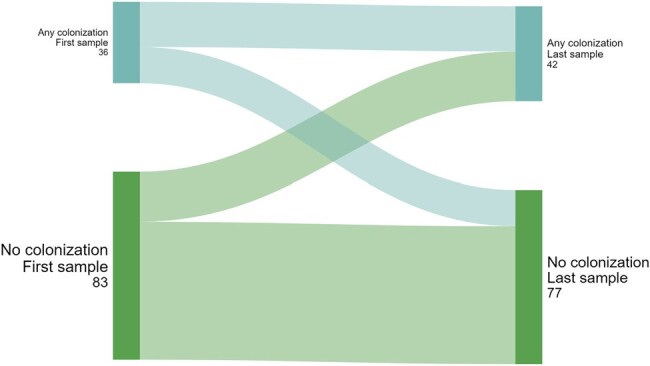
Changes in multi-drug resistant stool colonization status between first and last sample for 119 patients who had multiple samples.

**Conclusion:**

Over 40% of ICU patients were colonized with VRE, ESBL-E, and/or CRE, although colonization status commonly changed over time. Colonization status detected at one timepoint (e.g., ICU admission) may not reflect future states. More research is needed to identify factors that facilitate persistence and loss of colonization, and how this influences outcomes.

**Figure 3.**
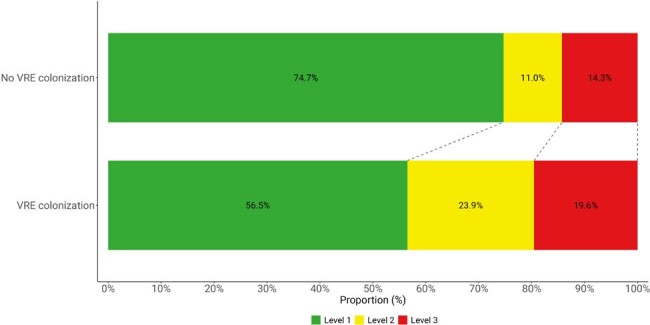
Desirability of outcome ranking (DOOR) levels comparing intensive care unit patients without (top bar) and with (bottom bar) VRE stool colonization.

**Disclosures:**

**All Authors**: No reported disclosures

